# Understanding and Supporting Attention Deficit Hyperactivity Disorder (ADHD) in the Primary School Classroom: Perspectives of Children with ADHD and their Teachers

**DOI:** 10.1007/s10803-022-05639-3

**Published:** 2022-07-01

**Authors:** Emily McDougal, Claire Tai, Tracy M. Stewart, Josephine N. Booth, Sinéad M. Rhodes

**Affiliations:** 1 Centre for Clinical Brain Sciences, Child Life and Health, University of Edinburgh, Edinburgh, United Kingdom; 2 Moray House School of Education and Sport, University of Edinburgh, Edinburgh, United Kingdom; 3 School of Psychology, University of Surrey, Guildford, United Kingdom

**Keywords:** ADHD, Child and teacher interviews, Strengths and difficulties, Learning strategies

## Abstract

Children with Attention Deficit Hyperactivity Disorder (ADHD) are more at risk for academic underachievement compared to their typically developing peers. Understanding their greatest strengths and challenges at school, and how these can be supported, is vital in order to develop focused classroom interventions. Ten primary school pupils with ADHD (aged 6–11 years) and their teachers (N = 6) took part in semi-structured interviews that focused on (1) ADHD knowledge, (2) the child’s strengths and challenges at school, and (3) strategies in place to support challenges. Thematic analysis was used to analyse the interview transcripts and three key themes were identified; classroom-general versus individual-specific strategies, heterogeneity of strategies, and the role of peers. Implications relating to educational practice and future research are discussed.

Characterised by persistent inattention, hyperactivity and impulsivity (APA, 2013), ADHD is a neurodevelopmental disorder thought to affect around 5% of children (Russell et al., 2014) although prevalence estimates vary (Sayal et al., 2018). Although these core symptoms are central to the ADHD diagnosis, those with ADHD also tend to differ from typically developing children with regards to cognition and social functioning (Coghill et al., 2014; Rhodes et al., 2012), which can negatively impact a range of life outcomes such as educational attainment and employment (Classi et al., 2012; Kuriyan et al., 2013). Indeed, academic outcomes for children with ADHD are often poor, particularly when compared with their typically developing peers (Arnold et al., 2020) but also compared to children with other neurodevelopmental disorders, such as autism (Mayes et al., 2020). Furthermore, children with ADHD can be viewed negatively by their peers. For example, Law et al. (2007) asked 11–12-year-olds to read vignettes describing the behaviour of a child with ADHD symptoms, and then use an adjective checklist to endorse those adjectives that they felt best described the target child. The four most frequently ascribed adjectives were all negative (i.e. ‘careless’, ‘lonely’, ‘crazy’, and ‘stupid’). These negative perceptions can have a significant impact on the wellbeing of individuals with ADHD, including self-stigmatisation (Mueller et al., 2012). There is evidence that teachers with increased knowledge of ADHD report more positive attitudes towards children with ADHD compared to those with poor knowledge (Ohan et al., 2008) and thus research that identifies the characteristics of gaps in knowledge is likely to be important in addressing stigma.

Previous research of teachers' ADHD knowledge is mixed, with the findings of some studies indicating that teachers have good knowledge of ADHD (Mohr-Jensen et al., 2019; Ohan et al., 2008) and others suggesting that their knowledge is limited (Latouche & Gascoigne, 2019; Perold et al., 2010). Ohan et al. (2008) surveyed 140 primary school teachers in Australia who reported having experience of teaching at least one child with ADHD. Teachers completed the ADHD Knowledge Scale which consisted of 20 statements requiring a response of either true or false (e.g. “A girl/boy can be appropriately labelled as ADHD and not necessarily be over-active*”).* They found that, on average, teachers answered 76.34% of items correctly, although depth of knowledge varied across the sample. Almost a third of the sample (29%) had low knowledge of ADHD (scoring less than 69%), with just under half of teachers (47%) scoring in the average range (scores of 70–80%). Only a quarter (23%) had “high knowledge” (scores above 80%) suggesting that knowledge varied considerably. Furthermore, Perold et al. (2010) asked 552 teachers in South Africa to complete the Knowledge of Attention Deficit Disorders Scale (KADDS) and found that on average, teachers answered only 42.6% questions about ADHD correctly. Responses of “don’t know” (35.4%) and incorrect responses (22%) were also recorded, indicating gaps in knowledge as well as a high proportion of misconceptions. Similar ADHD knowledge scores were reported in Latouche and Gascoigne’s (2019) study, who found that teachers enrolled into their ADHD training workshop in Australia had baseline KADDS scores of below 50% accuracy (increased to above 80% accuracy after training).

The differences in ADHD knowledge reported between Ohan et al. (2008) and the more recent studies could be due to the measures used. Importantly, when completing the KADDS, respondents can select a “don’t know” option (which receives a score of 0), whereas the ADHD Knowledge Scale requires participants to choose either true or false for each statement. The KADDS is longer, with a total of 39 items, compared to the 20-item ADHD Knowledge Scale, offering a more in-depth knowledge assessment. The heterogeneity of measures used within the described body of research is also highlighted within Mohr-Jensen et al. (2019) systematic review; the most frequently used measure (the KADDS) was only used by 4 out of the 33 reviewed studies, showing little consensus on the best way to measure ADHD knowledge. Despite these differences in measurement, the findings from most studies indicate that teacher ADHD knowledge is lacking.

Qualitative methods can provide rich data, facilitating a deeper understanding of phenomena that quantitative methods alone cannot reveal. Despite this, there are very few examples in the literature of qualitative methods being used to understand teacher knowledge of ADHD. In one example, Lawrence et al. (2017) interviewed fourteen teachers in the United States about their experiences of working with pupils with ADHD, beginning with their knowledge of ADHD. They found that teachers tended to focus on the external symptoms of ADHD, expressing knowledge of both inattentive and hyperactive symptoms. Although this provided key initial insights into the nature of teachers’ ADHD knowledge, only a small section of the interview schedule (one out of eight questions/topics) directly focused on ADHD knowledge. Furthermore, none of the questions asked directly about strengths, with answers focusing on difficulties. It is therefore difficult to determine from this study whether teachers are aware of strengths and difficulties outside of the triad of symptoms. A deeper investigation is necessary to fully understand what teachers know, and to identify areas for targeted psychoeducation.

Importantly, improved ADHD knowledge may impact positively on the implementation of appropriate support for children with ADHD in school. For example, Ohan et al. (2008) found that teachers with high or average ADHD knowledge were more likely to perceive a benefit of educational support services than those with low knowledge, and teachers with high ADHD knowledge were also more likely to endorse a need for, and seek out, those services compared to those with low knowledge. Furthermore, improving knowledge through psychoeducation may be important for improving fidelity to interventions in ADHD (Dahl et al., 2020; Nussey et al., 2013). Indeed, clinical guidelines recommend inclusion of psychoeducation in the treatment plan for children with ADHD and their families (NICE, 2018). Furthermore, Jones and Chronis-Tuscano (2008) found that educational ADHD training increased special education teachers’ use of behaviour management strategies in the classroom. Together, these findings suggest that understanding of ADHD may improve teachers’ selection and utilisation of appropriate strategies.

Child and teacher insight into strategy use in the classroom on a practical, day-to-day level may provide an opportunity to better understand how different strategies might benefit children, as well as the potential barriers or facilitators to implementing these in the classroom. Previous research with teachers has shown that aspects of the physical classroom can facilitate the implementation of effective strategies for autistic children, for example to support planning with the use of visual timetables (McDougal et al., 2020). Despite this, little research has considered the strategies that children with ADHD and their teachers are using in the classroom to support their difficulties and improve learning outcomes. Moore et al. (2017) conducted focus groups with UK-based educators (N = 39) at both primary and secondary education levels, to explore their experiences of responding to ADHD in the classroom, as well as the barriers and facilitators to supporting children. They found that educators mostly reflected on general inclusive strategies in the classroom that rarely targeted ADHD symptoms or difficulties specifically, despite the large number of strategies designed to support ADHD that are reported elsewhere in the literature (DuPaul et al., 2012; Richardson et al., 2015). Further to this, when interviewing teachers about their experiences of teaching pupils with ADHD, Lawrence et al. (2017) specifically asked about interventions or strategies used in the classroom with children with ADHD. The reported strategies were almost exclusively behaviourally based, for example, allowing children to fidget or move around the classroom, utilising rewards, using redirection techniques, or reducing distraction. This lack of focus on cognitive strategies is surprising, given the breadth of literature focusing on the cognitive difficulties in ADHD (e.g. Coghill, et al., 2014; Gathercole et al., 2018; Rhodes et al., 2012). Furthermore, to our knowledge research examining strategy use from the perspective of children with ADHD themselves, or strengths associated with ADHD, is yet to be conducted.

Knowledge and understanding of ADHD in children with ADHD has attracted less investigation than that of teachers. In a Canadian sample of 8- to 12-year-olds with ADHD (N = 29), Climie and Henley (2018) found that ADHD knowledge was highly varied between children; scores on the Children ADHD Knowledge and Opinions Scale ranged from 5 to 92% correct (M = 66.53%, SD = 18.96). The authors highlighted some possible knowledge gaps, such as hyperactivity not being a symptom for all people with ADHD, or the potential impact upon social relationships, however the authors did not measure participant’s ADHD symptoms, which could influence how children perceive ADHD. Indeed, Wiener et al (2012) has shown that children with ADHD may underestimate their symptoms. If this is the case, it would also be beneficial to investigate their understanding of their own strengths and difficulties, as well as of ADHD more broadly. Furthermore, if children do have a poor understanding of ADHD, they may benefit from psychoeducational interventions. Indeed, in their systematic review Dahl et al. (2020) found two studies in which the impact of psychoeducation upon children’s ADHD knowledge was examined, both of which reported an increase in knowledge as a consequence of the intervention. Understanding the strengths and difficulties of the child, from the perspective of the child and their teacher, will also allow the design of interventions that are individualised, an important feature for school-based programmes (Richardson et al., 2015). Given the above, understanding whether children have knowledge of their ADHD and are aware of strategies to support them would be invaluable.

Teacher and child knowledge of ADHD and strategies to support these children is important for positive developmental outcomes, however there is limited research evidence beyond quantitative data. Insights from children and teachers themselves is particularly lacking and the insights which are available do not always extend to understanding strengths which is an important consideration, particularly with regards to implications for pupil self-esteem and motivation. The current study therefore provides a vital examination of the perspectives of both strengths and weaknesses from a heterogeneous group of children with ADHD and their teachers. Our sample reflects the diversity encountered in typical mainstream classrooms in the UK and the matched pupil-teacher perspectives enriches current understandings in the literature. Specifically, we aimed to explore (1) child and teacher knowledge of ADHD, and (2) strategy use within the primary school classroom to support children with ADHD. This novel approach, from the dual perspective of children and teachers, will enable us to identify potential knowledge gaps, areas of strength, and insights on the use of strategies to support their difficulties.

## Method

### Participants

Ten primary school children (3 female) aged 7 to 11 years (M = 8.7, SD = 1.34) referred to Child and Adolescent Mental Health Services (CAMHS) within the NHS for an ADHD diagnosis were recruited to the study. All participant characteristics are presented in Table 1. All children were part of the Edinburgh Attainment and Cognition Cohort and had consented to be contacted for future research. Children who were under assessment for ADHD or who had received an ADHD diagnosis were eligible to take part. Contact was established with the parent of 13 potential participants. Two had undergone the ADHD assessment process with an outcome of no ADHD diagnosis and were therefore not eligible to take part, and one could not take part within the timeframe of the study. The study was approved by an NHS Research Ethics Committee and parents provided informed consent prior to their child taking part. Co-occurrences data for all participants was collected as part of a previous study and are reported here for added context. All of the children scored above the cut-off (T-score > 70) for ADHD on the Conners 3^rd^ Edition Parent diagnostic questionnaire (Conners, 2008). The maximum possible score for this measure is 90. At the point of interview, seven children had received a diagnosis of ADHD, two children were still under assessment, and one child had been referred for an ASD diagnosis (Table 1). The ADHD subtype of each participant was not recorded, however all children scored above the cut-off for both inattention (M = 87.3, SD = 5.03) and hyperactivity (M = 78.6, SD = 5.8) which is indicative of ADHD combined type. Use of stimulant medication was not recorded at the time of interview.

**Table 1:** Participant demographics

Pseudonym	Child’s age	Child’s gender	Diagnosis at time of interview	Conners’-P Inattention	Conners’-P Hyperactivity	AQ-10 total	M-ABC2 zone	SIMD	Teaching experience (years)	Perceived experience teaching children with ADHD
Henry	9	M	Under assessment	90	90	3	Red	4	13	Moderate
Jay	7	M	ADHD	74	72	4	Red	1	23	Moderate
Felicity	11	F	Under assessment	88	90	1	Red	3	N/A	N/A
Paige	8	F	ADHD	90	90	4	Red	5	20	Moderate
Eric	10	M	ADHD	90	90	3	Green	1	5	Small-to-moderate
Rory	9	M	ADHD	90	90	3	Amber	5	N/A	N/A
Luna	8	F	Under assessment	90	90	2	Green	5	1	Small
Nathan	8	M	ADHD	86	84	8*	Red	1	9	Significant
Finn	10	M	ADHD	85	90	4	Red	2	N/A	N/A
Niall	7	M	ADHD	90	90	4	Red	1	N/A	N/A

Following the child interview and receipt of parental consent, each child’s school was contacted to request their teacher’s participation in the study. Three teachers could not take part within the timeframe of the study, and one refused to take part. Six teachers (all female) were successfully contacted and gave informed consent to participate.

Due to the increased likelihood of co-occurring diagnoses in the target population, we also report Autism Spectrum Disorder (ASD) symptoms and Developmental Co-ordination Disorder (DCD) symptoms using the Autism Quotient 10-item questionnaire (AQ-10; Allison et al., 2012) and Movement ABC-2 Checklist (M-ABC2; Henderson et al., 2007) respectively, both completed by the child’s parent.

Scores of 6 and above on the AQ-10 indicates referral for diagnostic assessment for autism is advisable. All but one of the participants scored below the cut-off on this measure (M = 3.6, SD = 1.84).

The M-ABC2 checklist categorises children as scoring green, amber or red based on their scores. A green rating (up to the 85th percentile) indicates no movement difficulty, amber ratings (between 85 and 95th percentile) indicate risk of movement difficulty, and red ratings (95th percentile and above) indicate high likelihood of movement difficulty. Seven of the participants received a red rating, one an amber rating, and two green ratings.

Socioeconomic status (SES) is also known to impact educational outcomes, therefore the SES of each child was calculated using the Scottish Index of Multiple Deprivation (SIMD), which is an area-based measure of relative deprivation. The child’s home postcode was entered into the tool which provided a score of deprivation on a scale of 1 to 5. A score of 1 is given to the 20% most deprived data zones in Scotland, and a score of 5 indicates the area was within the 20% least deprived areas.

### Semi-Structured Interview

The first author, who is a psychologist, conducted interviews with each participant individually, and then a separate interview with their teacher. This was guided by a semi-structured interview schedule (see Appendix A, Appendix B) developed in line with our research questions, existing literature, and using authors (T.S. and J.B.) expertise in educational practice. The questions were adapted to be relevant for the participant group. For example, children were asked “If a friend asked you to tell them what ADHD is, what would you tell them?” and teachers were asked, “What is your understanding of ADHD or can you describe a typical child with ADHD?”. The schedule comprised two key sections for both teachers and children. The first section focused on probing the participant’s understanding and knowledge of ADHD broadly. The second section focused on the participating child’s academic and cognitive strengths and weaknesses, and the strategies used to support them. Interviews with children took place in the child’s home and lasted between 19 and 51 min (M = 26.3, SD = 10.9). Interviews with teachers took place at their school and were between 28 and 50 min long (M = 36.5, SD = 7.61). Variation in interview length was mostly due to availability of the participant and/or age of the child (i.e. interviews with younger children tended to be shorter). All interviews were recorded on an encrypted voice recorder and transcribed by the first author prior to data analysis. Pseudonyms were randomly generated for each child to protect anonymity.

### Analysis

Reflexive thematic analysis was used to analyse the data (Braun & Clarke, 2019). This flexible approach allows the data to drive the analysis, putting the participant at the centre of the research and placing high value on the experiences and perspectives of individual participants (Braun & Clarke, 2006). The six phases of reflexive thematic analysis as outlined by Braun and Clarke were followed: (1) familiarisation, (2) generating codes, (3) constructing themes, (4) revising themes, (5) defining themes, (6) producing the report. Due to the exploratory nature of this study, bottom-up inductive coding was used. Two of the authors (E.M. and C.T.) worked collaboratively to construct and subsequently define the themes using the process described above. More specifically, one author (E.M.) generated codes, with support from another author (C.T.). Collated codes and data were then abstracted into potential themes, which were reviewed and refined using relevant literature, as well as within the wider context of the data. This process continued until all themes were agreed upon.

In the first part of the analysis, focus was placed on summarising the participants’ understanding of ADHD, as well as what they thought their biggest strengths and challenges were at school. Following this, an in-depth analysis of the strategies used in the classroom was conducted, taking into account the perspective of both teachers and children, aiming to generate themes from the data.

## Results

### Knowledge of ADHD

Children and teachers were asked about their knowledge of ADHD. When asked if they had ever heard of ADHD, the majority of children said yes. Some of the children could not explain to the interviewer what ADHD was or responded in a way that suggested a lack of understanding (*“it helps you with skills”*– Niall, 7 years; “*Well it’s when you can’t handle yourself and you’re always crazy and you can just like do things very fast”—*Nathan, 8 years). Very few of the children were able to elaborate accurately on their understanding of ADHD, which exclusively focused on inattention. For example, Paige (8 years) said “*its’ kinda like this thing that makes it hard to concentrate*” and Finn (10 years) said “*they get distracted more just in different ways that other people would*”. This suggests that children with ADHD may lack or have a limited awareness or understanding of their diagnosis.

When asked about their knowledge of ADHD, teachers tended to focus on the core symptoms of ADHD. All teachers directly mentioned difficulties with attention, focus or concentration, and most directly or indirectly referred to hyperactivity (e.g. moving around, being in “*overdrive*”). Most teachers also referred to social difficulties as a feature of ADHD, including not following social rules, reacting inappropriately to other children and appearing to lack empathy, which they suggested could be linked to impulsivity. For example, “*reacting in social situations where perhaps other children might not react in a similar way”* (Paige’s teacher) and “*They can react really really quickly to things and sometimes aggressively”* (Eric’s teacher). Although no teachers directly mentioned cognitive difficulties, some referred to behaviours indicative of cognitive difficulties, for example, “*they can’t store a lot of information at one time”* (Eric’s teacher) and, “*it’s not just the concentration it’s the amount they can take in at a time as well”* (Nathan’s teacher), which may reflect processing or memory differences. Heterogeneity was mentioned, in that ADHD can mean different things for different children (e.g., “*I think ADHD differs from child to child and I think that’s really important”*—Nathan’s teacher). Finally, academic difficulties as a feature of ADHD were also mentioned (e.g., “*a child… who finds some aspects of school life, some aspects of the curriculum challenging*”—Jay’s teacher).

### Strengths

After being asked to give a general description of ADHD, each child was asked about their own strengths at school and teachers were also asked to reflect on this topic for the child taking part.

When asked what they like most about school, children often mentioned art or P.E. as their preferred subjects. A small number of children said they enjoyed maths or reading, but this was not common and the majority described these subjects as a challenge or something they disliked. There was also clear link between the aspects of school children enjoyed, and what they perceived to be a strength for them. For example, when asked what he liked about school, Eric (10 years) said, “*Math, I’m pretty good at that”,* or when later asked what they were good at, most children responded with the same answers they gave when asked what they liked about school. It is interesting to note that subjects such as art or P.E. generally have a different format to more traditionally academic subjects such as maths or literacy. Indeed, Felicity (11 years) said, “*I quite like art and drama because there’s not much reading…and not really too much writing in any of those”*. Children also tended to mention the non-academic aspects of school, such as seeing their friends, or lunch and break times.

Teachers’ descriptions of the children’s strengths were much more variable compared to strengths mentioned by children. Like the children, teachers tended to consider P.E and artistic activities to be a strength for the child with ADHD. Multiple teachers referred to the child having a good imagination and creative skills. For example, “*she’s a very imaginative little girl, she has a great ability to tell stories and certainly with support write imaginative stories”* (Paige’s teacher)*.* Teachers referred to other qualities or characteristics of the child as strengths, although these varied across teachers. These included openness, both socially but also in the context of willingness to learn or being open to new challenges, being a hard worker, or an enjoyable person to be around (e.g., “*he is the loveliest little boy, I’ve got a lot of time for [Nathan]. He makes me smile every day, you know, he just comes out with stuff he’s hilarious”—*Nathan’s teacher). The most noticeable theme that emerged from this data was that when some teachers began describing one of the child’s strengths, it was suffixed with a negative. For example, Henry’s teacher said, “*He’s got a very good imagination, his writing- well not so much the writing of the stories, he finds writing quite a challenge, but his verbalising of ideas he’s very imaginative”.* This may reflect that while these children have their own strengths, these can be limited by difficulties. Indeed, Paige’s teacher said, “*I think she’s a very able little girl without a doubt, but there is a definite barrier to her learning in terms of her organisation, in terms of her focus”*, which reinforces this notion.

### Challenges

Children were asked directly about what they disliked about school, and what they found difficult. Children tended to focus more on specific subjects, with maths and aspects of literacy being the most frequently mentioned of these. Children referred to difficulties with or a dislike for reading, writing and/or spelling activities, for example, Rory (9 years) said “*Well I suppose spelling because … sometimes we have to do some boring tasks like we have to write it out three times then come up with the sentence for each one which takes forever and it’s hard for me to think of the sentences if I’m not ready”*. Linking this with known cognitive difficulties in ADHD, it is interesting to note that both memory and planning are implicated in this quote from Rory about finding spelling challenging. In terms of writing, children referred to both the physical act of writing (e.g., “*probably writing cause sometimes I forget my finger spaces*”—Paige, 8 years; “*[writing the alphabet is] too hard… like the letters joined together … [and] I make mistakes”*—Jay, 7 years) as well as the planning associated with writing a longer piece of work (e.g. “*when I run out of ideas for it, it’s really hard to think of some more so I don’t usually get that much writing done*”—Rory (9 years)*.*

Aside from academic subjects, several children referred to difficulties with focus or attention (e.g. “*when I find it hard to do something I normally kind of just zone out*”—Felicity, 11 years, “*probably concentrating sometimes*”—Rory, 9 years), but boredom was also a common and potentially related theme (e.g. “*Reading is a bit hard though … it just sometimes gets a bit boring”*—Finn, 10 years, “*I absolutely hate maths … ‘cause it’s boring*”—Paige, 8 years). It could be that children with ADHD find it more difficult to concentrate during activities they find boring. Indeed, when Jay (7 years) was asked how it made him feel when he found something boring, he said “*it made me not do my work*”. Some children also alluded to the social difficulties faced at school, which included bullying and difficulties making friends (e.g. “*just making all kind of friends [is difficult] ‘cause the only friend that I’ve got is [name redacted]*”—Nathan, 8 years; “*sometimes finding a friend to play with at break time [is difficult]*” – Paige, 8 years; “*there’s a lot of people in my school that they bully me”*—Eric, 10 years).

When asked what they thought were the child’s biggest challenges at school, teachers' responses were relatively variable, although some common themes were identified. As was the case for children, teachers reflected on difficulties with attention, which also included being able to sit at the table for long periods of time (e.g. “*I would say he struggles the most with sitting at his table and focusing on one piece of work*”—Henry’s teacher). Teachers did also mention difficulties with subjects such as maths and literacy, although this varied from child to child, and often they discussed these in the context of their ADHD symptom-related difficulties. For example, Eric’s teacher said, “*we’ve struggled to get a long piece of writing out of him because he just can’t really sit for very long*”. This quote also alludes to difficulties with evaluating the child’s academic abilities, due to their ADHD-related difficulties, which was supported by other teachers (e.g. “*He doesn’t particularly enjoy writing and he’s slow, very slow. And I don’t know if that’s down to attention or if that’s something he actually does find difficult to do*” —Henry’s teacher). Furthermore, some teachers reflected on the child’s confidence as opposed to a direct academic difficulty. For example, Luna’s teacher said, “*I think it’s she lacks the confidence in maths and reading like the most*” and later, elaborated with “*she’ll be like “I can’t do it” but she actually can. Sometimes she’s … even just anxious at doing a task where she thinks … she might not get it. But she does, she’s just not got that confidence”.*

Teachers also commonly mentioned social difficulties, and referred to these difficulties as a barrier to collaborative learning activities (e.g. “*he doesn’t always work well with other people and other people can get frustrated”*—Henry’s teacher; “*[during] collaborative group work [Paige] perhaps goes off task and does things she shouldn’t necessarily be doing and that can cause friction within the group”*—Paige’s teacher). Teachers also mentioned emotion regulation, mostly in relation to the child’s social difficulties. For example, Eric’s teacher said “*I think as well he does still struggle with his emotions like getting angry very very quickly, and being very defensive when actually he’s taken the situation the wrong way”*, which suggests that the child’s difficulty with regulating emotions may impact on their social relationships.

### Strategy Use in the Classroom

Strategies to support learning fell into one of four categories: concrete or visual resources, information processing, seating and movement, and support from or influence of others. Examples of codes included in each of these strategy categories are presented in Table 2.

**Table 2:** Strategy categories identified and example codes

Strategy category	Example codes
Concrete or visual resources	Visual timetable; visual story prompts; instructions written on board; times table squares; number lines; manipulatives; timers
Information processing and cognition	Shorter instructions; prompting for attention; repetition of instructions; regular breaks; reminders; chunking day or tasks; times table songs; routine
Seating and movement	Individual table; location in classroom; allowing movement; movement breaks; fidget cushion; fidget toys; resources on desk
Support from and influence of others	Small group or paired work; ideas from peers; praise; reward; understanding the child; using child’s name; write first sentence for child

Concrete or visual resources were the most commonly mentioned type of strategy by teachers and children, referring to the importance of having physical representations to support learning. Teachers spoke about the benefit of using visual aids (e.g. “*I think [Henry] is quite visual so making sure that there is visual prompts and clues and things like that to help him*”—Henry’s teacher), and teachers and children alluded to these resources supporting difficulties with holding information in mind. For example, when talking about the times table squares he uses, Rory said “*sometimes I forget which one I’m on…and it’s easier for me to have my finger next to it than just doing it in my head because sometimes I would need to start doing it all over again*”.

Seating and movement were also commonly mentioned, which seemed to be specific to children with ADHD in that it was linked to inattention and hyperactivity symptoms. For example, teachers referred to supporting attention or avoiding distraction by the positioning of a child’s location in the classroom (e.g. “*he’s so easily distracted, so he has an individual desk in the room and he’s away from everyone else because he wasn’t coping at a table [and] he’s been so much more settled since we got him an individual desk”*—Eric’s teacher). Some teachers also mentioned the importance of allowing children to move around the room where feasible, as well as giving them errands to perform as a movement break (e.g. “*if I need something from the printer, [Nathan] is gonna go for it for me…because that’s down the stairs and then back up the stairs so if I think he’s getting a bit chatty or he’s not focused I’ll ask him to go and just give him that break as well”*—Nathan’s teacher). Children also spoke about these strategies but didn’t necessarily describe why or how these strategies help them.

Information processing and cognitive strategies included methods that supported children to process learning content or instructions. For example, teachers frequently mentioned breaking down tasks or instructions into more manageable chunks (e.g. “*with my instructions to [Eric] I break them down*…*I’ll be like “we’re doing this and then we’re doing this” whereas the whole class wouldn’t need that*”—Eric’s teacher). Teachers and children also mentioned using memory strategies such as songs, rhymes or prompts. For example, Jay’s teacher said, “*if I was one of the other children I could see why it would be very distracting but he’s like he’s singing to himself little times table songs that we’ve been learning in class”*, and Paige (8 years) referred to using mnemonics to help with words she struggles to spell, “*I keep forgetting [the word] because. But luckily we got the story big elephants can always understand little elephants [which helps because] the first letter of every word spells because”*.

Both groups of participants mentioned support from and influence of others, and referred to working with peers, the teacher–child relationship, and one-to-one teaching. Peer support was a common theme across the data and is discussed in more detail in the thematic analysis findings, where teachers and children referred to the importance of the role of peers during learning activities. Understanding the child well and adapting to them was also seen as important, for example, Luna’s teacher said, “*with everything curricular [I] try and have an art element for her, just so I know it’ll engage her [because] if it’s like a boring old written worksheet she’s not gonna do it unless you’re sitting beside her and you’re basically telling her the answers”*. As indicated in this quote, teachers also referred to the effectiveness of one-to-one or small group work with the child (e.g. “*when somebody sits beside her and explains it, and goes “come on [Paige] you know how to do this, let’s just work through a couple of examples”… her focus is generally better*” – Paige’s teacher), however this resource is not always available (e.g. “*I’d love for someone to be one-to-one with [Luna] but it’s just not available, she doesn’t meet that criteria apparently*” – Luna’s teacher). Children also referred to seeking direct support from their teacher (e.g. “if I can’t get an idea of what I’m doing then I ask the teacher for help” – Paige, 8 years), but were more likely to mention seeking support from their peers than the teacher.

### Thematic Analysis

In addition to summarising the types of strategies that teachers and children reported using in the classroom, the data were also analysed using thematic analysis to generate themes. These are now presented. The theme names, definitions, and example quotes for each theme are presented in Table 3.

**Table 3:** Themes, theme definitions and example quotes

Theme	Definition	Example quotes
Classroom-general versus individual-specific strategies	Some strategies used in the classroom are available for everyone, but are particularly useful for children with ADHD. Other strategies are in place for specific children. Children have their own strategies that teachers may not be aware of	‘*there’s also a morning routine and listing down what’s to be done and where it’s to go … it’s very general for the class but again it’s located near her’* – Paige’s teacher *‘if she’s wandering around the classroom or she’s sitting on a table, I don’t let other kids do that, but as long as she’s listening, it’s fine [with me]’* – Luna’s teacher *‘if you just bring [a fidget toy] in without permission [the teacher will] just take it off of you, so it has to be something that’s not too big’* – Henry
Heterogeneity of strategies	The efficacy of strategies varies between and within children. A strategy helpful for one child may not be helpful for another. The effectiveness of a strategy may vary over time or between contexts	‘*some [strategies] will work for the majority of the children and some just don’t seem to work for any of them*’ – Jay’s teacher *‘some things have worked and then stopped working, so I think we’re constantly adapting and changing what we’re doing*’ – Eric’s teacher
The role of peers	Peers play an important role in supporting children with ADHD, but the way they are viewed differs between teachers and children	‘*giving him a role and making sure that there’s people in the group that would encourage him to fulfil his role [is helpful]*’ – Henry’s teacher ‘*some things I might not know and the children might help me give ideas*’ – Niall

#### Theme 1: Classroom-General Versus Individual-Specific Strategies

During the interviews, teachers spoke about strategies that they use as part of their teaching practice for the whole class but that are particularly helpful for the child/children with ADHD. These tended to be concrete or visual resources that are available in the classroom for anyone, for example, a visual timetable or routine checklist (e.g. “*there’s also a morning routine and listing down what’s to be done and where it’s to go … it’s very general for the class but again it’s located near her”*—Paige’s teacher).

Teachers also mentioned using strategies that have been implemented specifically for that child, and these strategies tended to focus on supporting attention. For example, Nathan’s teacher spoke about the importance of using his name to attract his attention, “*maybe explaining to the class but then making sure that I’m saying “[Nathan], you’re doing this”, you know using his name quite a lot so that he knows it’s his task not just the everybody task*”, and this was a strategy that multiple teachers referred to using with the individual child and not necessarily for other children. Other strategies to support attention with a specific child also tended to be seating and movement related, such as having an individual desk or allowing them to fidget. For example, Luna’s teacher said, “*she’s a fidgeter so she’ll have stuff to fidget with … [and] even if she’s wandering around the classroom or she’s sitting on a table, I don’t let other kids do that, but as long as she’s listening, it’s fine [with me]”*.

Similar to teachers, children spoke about strategies or resources that were in place for them specifically as well as about general things in the classroom that they find helpful. That said, it was less common for children to talk about *why* particular strategies were in place for them and how they helped them directly.

In addition to recognising strategies that teachers had put in place for them, children also referred to using their own strategies in the classroom. The most frequently mentioned strategy was fidgeting, and although some of the younger children spoke about having resources available in the classroom for fidgeting, some of the older children referred to using their own toy or an object that was readily available to them but not intended for fidgeting. For example, Finn (10 years) and Rory (9 years) both spoke about using items from their pencil case to fiddle with, and explained that this would help them to focus. (“*Sometimes I fidget with something I normally just have like a pencil holder under the table moving about … [and] it just keeps my mind clear and not from something else*”—Rory; “*Sometimes I fiddle with my fingers and that sometimes helps, but if not I get one of my coloured pencils and have a little gnaw on it because that actually takes my mind off some things and it’s easier for me to concentrate when I have something to do*”—Finn). Henry (9 years) spoke about being secretive with his fidgeting as it was not permitted in class, “*if you just bring [a fidget toy] in without permission [the teacher will] just take it off of you, so it has to be something that’s not too big. I bring in a little Lego ray which is just small enough that she won’t notice*”. Although some teachers did mention having fidget toys available, not all teachers seemed to recognise the importance of this for the child, and some children viewed fidgeting as a behaviour they should hide from the teacher.

Another strategy mentioned uniquely by children was seeing their peers as a resource for ideas or information. This is discussed in more detail in *Theme 3—The role of peers*, but reinforces the notion that children also develop their own strategies, independently from their teacher, rather than relying only on what is made available to them.

#### Theme 2: Heterogeneity of Strategies

Teachers spoke about the need for a variety of strategies in the classroom, for two reasons: (1) that different strategies work for different children (e.g. “*some [strategies] will work for the majority of the children and some just don’t seem to work for any of them*”—Jay’s teacher), and (2) what works for a child on one occasion may not work consistently for the same child (e.g. “*I think it’s a bit of a journey with him, and some things have worked and then stopped working, so I think we’re constantly adapting and changing what we’re doing*”—Eric’s teacher). One example of both of these challenges of strategy use came from Luna’s teacher, who spoke about using a reward chart with Luna and another child with ADHD, “*[Luna] and another boy in my class [with ADHD] both had [a reward chart]… but I think whereas the boy loved his and still loves his, she was getting a bit “oh I’m too cool for this” or that sort of age… so I stopped doing that for her and she’s not missing that at all”*. These quotes demonstrate that strategies can work differently for different children, highlighting the need for a variety of strategies for teachers to access and trial with children.

Some children also referred to the variability of whether a strategy was helpful or not; for example, Henry (9 years) said that he finds it helpful to fidget with a toy but that sometimes it can distract him and prevent him from listening to the teacher. He said, “*Well, [the fidget toy] helps but it also gets me into trouble when the teacher spots me building it when I’m listening…but then sometimes I might not listen in maths and [use the fidget toy] which might make it worse”.* This highlights that both children and teachers might benefit from support in understanding the contexts in which to use particular strategies, as well as why they are helpful from a psychological perspective.

For teachers, building a relationship with and understanding the child was also highly important in identifying strategies that would work. Luna’s teacher reflected upon the difference in Luna’s behaviour at the start of the academic year, compared to the second academic term, “*at the start of the year, we would just clash the whole time. I didn’t know her, she didn’t know me … and then when we got that bond she was absolutely fine so her behaviour has got way better*”. Eric’s teacher also reflected on how her relationship with Eric had changed, particularly after he received his diagnosis of ADHD, “*I think my approach to him has completely changed. I don’t raise my voice, I speak very calmly, I give him time to calm down before I even broach things with him. I think our relationship’s just got so much better ‘cause I kind of understand … where he’s coming from*”. She also said, “*it just takes a long time to get to know the child and get to know what works for them and trialling different things out*”, which demonstrates that building a relationship with and understanding the child can help to identify the successful strategies that work with different children.

#### Theme 3: The Role of Peers

Teachers and children spoke about the role of the child’s peers in their learning. Teachers talked about the benefit of partnering the child with good role models (e.g. “*I will put him with a couple of good role models and a couple of children who are patient and who will actually maybe get on with the task, and if [Jay] is not on task or not on board with what they’re doing at least he’s hearing and seeing good behaviour*”—Jay’s teacher), whereas children spoke more about their peers as a source of information, idea generation, or guidance on what to do next. For example, when asked what he does to help him with his writing, Henry (9 years) said, “*[I] listen to what my partner’s saying… my half of the table discuss what they’re going to do so I can literally hear everything they’re doing and steal some of their ideas*”. Henry wasn’t the only child to use their peers as a source of information, for example, Niall (7 years) said, “*I prefer working with the children because some things I might not know and the children might help me give ideas*”, and with a more specific example, Rory (9 years) said, “*somebody chose a very good character for their bit of writing, and I was like “I think I might choose that character”, and somebody else said “my setting was going to be the sea”, and I chose that and put that in a tiny bit of my story*”.

Some children also spoke about getting help from their peers in other ways, particularly when completing a difficult task. Paige (8 years) said, “*if the question isn’t clear I try and figure it out, and if I can’t figure it out then… don’t tell my teacher this but I sometimes get help from my classmates*”, which suggests some guilt associated with asking for help from her peers. This could be related to confidence and self-esteem, which teachers mentioned as a difficulty for some children with ADHD. In some instances, children felt it necessary to directly copy their peers’ work; for example, Nathan (8 years) spoke about needing a physical resource (i.e. “*fuzzies*”) to complete maths problems, but that when none were available he would “*just end up copying other people*”. This could also be related to a lack of confidence, as he may feel as though he may not be able to complete the task on his own. Indeed, Nathan’s teacher mentioned that when he is given the option to choose a task from different difficulty levels, Nathan would typically choose something easier, and that it was important to encourage him to choose something more difficult to build his confidence, “*I quite often say to him “come on I think you can challenge yourself” and [will] use that language”.*

Peers clearly play an important role for the children with ADHD, and this is recognised both by the children themselves, and by their teachers. Teachers also mentioned that children with ADHD respond well to one-to-one learning with staff, indicating that it is important for these children to have opportunities to learn in different contexts: whole classroom learning, small group work and one-to-one.

## Discussion

In this study, a number of important topics surrounding ADHD in the primary school setting were explored, including ADHD knowledge, strengths and challenges, and strategy use in the classroom, each of which will now be discussed in turn before drawing together the findings and outlining the implications.

### ADHD Knowledge

Knowledge of ADHD varied between children and their teachers. Whilst most of the children claimed to have heard of ADHD, very few could accurately describe the core symptoms. Previous research into this area is limited, however this finding supports Climie and Henley’s (2018) finding that children’s knowledge of ADHD can be limited. By comparison, all of the interviewed teachers had good knowledge about the core ADHD phenotype (i.e. in relation to diagnostic criteria) and some elaborated further by mentioning social difficulties or description of behaviours that could reflect cognitive difficulties. This supports and builds further upon existing research into teachers’ ADHD knowledge, demonstrating that although teachers understanding may be grounded in a focus upon inattention and hyperactivity, this is not necessarily representative of the range of their knowledge. By interviewing participants about their ADHD knowledge, as opposed to asking them to complete a questionnaire as previous studies have done (Climie & Henley, 2018; Latouche & Gascoigne, 2019; Ohan et al., 2008; Perold et al., 2010), the present study has demonstrated the specific areas of knowledge that should be targeted when designing psychoeducation interventions for children and teachers, such as broader aspects of cognitive difficulties in executive functions and memory. Improving knowledge of ADHD in this way could lead to increased positive attitudes and reduction of stigma towards individuals with ADHD (Mueller et al., 2012; Ohan et al., 2008), and in turn improving adherence to more specified interventions (Bai et al., 2015).

### Strengths and Challenges

A range of strengths and challenges were discussed, some of which were mentioned by both children and teachers, whilst others were unique to a particular group. The main consensus in the current study was that art and P.E. tended to be the lessons in which children with ADHD thrive the most. Teachers elaborated on this notion, speaking about creative skills, such as a good imagination, and that these skills were sometimes applied in other subjects such as creative writing in literacy. Little to no research has so far focused on the strengths of children with ADHD, therefore these findings identify important areas for future investigation. For example, it is possible that these strengths could be harnessed in educational practice or intervention.

Although a strength for some, literacy was commonly mentioned as a challenge by both groups, specifically in relation to planning, spelling or the physical act of writing. Previous research has repeatedly demonstrated that literacy outcomes are poorer for children with ADHD compared to their typically developing peers (DuPaul et al., 2016; Mayes et al., 2020), however in these studies literacy tended to be measured using a composite achievement score, where the nuance of these difficulties can be lost. Furthermore, in line with a recent systematic review and meta-analysis (McDougal et al., 2022) the present study’s findings suggest that cognitive difficulties may contribute to poor literacy performance in ADHD. This issue was not unique to literacy, however, as teachers also spoke about academic challenges in the context of ADHD symptoms being a barrier to learning, such as finding it difficult to remain seated long enough to complete a piece of work. Children also raised this issue of engagement, who referred to the most challenging subjects being ‘boring’ for them. This link between attention difficulties and boredom in ADHD has been well documented (Golubchik et al., 2020). The findings here demonstrate the need for further research into the underlying cognitive difficulties leading to academic underachievement.

Both children and teachers also mentioned social and emotional difficulties. Research has shown that many different factors may contribute to social difficulties in ADHD (for a review see Gardner & Gerdes, 2015), making it a complex issue to disentangle. That said, in the current study teachers tended to attribute the children’s relationship difficulties to behaviour, such as reacting impulsively in social situations, or going off task during group work, both of which could be linked to ADHD symptoms. Despite these difficulties, peers were also considered a positive support. This finding adds to the complexity of understanding social difficulties for children with ADHD, demonstrating the necessity and value of further research into this key area.

### Strategy Use in the Classroom

The three key themes of *classroom-general versus individual-specific strategies*, *heterogeneity of strategies* and *the role of peers* were identified from the interview transcripts with children and their teachers. Within the first theme, classroom-general versus individual-specific strategies, it was clear that teachers utilise strategies that are specific to the child with ADHD, as well as strategies that are general to the classroom but that are also beneficial to the child with ADHD. Previously, Moore et al. (2017) found that teachers mostly reflected on using general inclusive strategies, rather than those targeted for ADHD specifically, however the methods differ from the current study in two key ways. Firstly, Moore et al.’s sample included secondary and primary school teachers, for whom the learning environment is very different. Secondly, focus groups were used as opposed to interviews where the voices of some participants can be lost. The merit of the current study is that children were also interviewed using the same questions as teachers; we found that children also referred to these differing types of strategies, and reported finding them useful, suggesting that the reports of teachers were accurate. Interestingly, children also mentioned their own strategies that teachers did not discuss and may not have been aware of. This finding highlights the importance of communication between the child and the teacher, particularly when the child is using a strategy considered to be forbidden or discouraged, for example copying a peer’s work or fidgeting with a toy. This communication would provide an understanding of what the child might find helpful, but more importantly identify areas of difficulty that may need more attention. Further to this, most strategies specific to the child mentioned by teachers aimed to support attention, and few strategies targeted other difficulties, particularly other aspects of cognition such as memory or executive function, which supports previous findings (Lawrence et al., 2017). The use of a wide range of individualised strategies would be beneficial to support children with ADHD.

Similarly, the second theme, *heterogeneity of strategies*, highlighted that some strategies work with some children and not others, and some strategies may not work for the same child consistently. Given the benefit of a wide range of strategy use, for both children with ADHD and their teachers, the development of an accessible tool-kit of strategies would be useful. Importantly, and as recognised in this second theme, knowing the individual child is key to identifying appropriate strategies, highlighting the essential role of the child’s teacher in supporting ADHD. Teachers mostly spoke about this in relation to the child’s interests and building rapport, however this could also be applied to the child’s cognitive profile. A tool-kit of available strategies and knowledge of which difficulties they support, as well as how to identify these difficulties, would facilitate teachers to continue their invaluable support for children and young people with ADHD. This links to the importance of psychoeducation; as previously discussed, the teachers in our study had a good knowledge of the core ADHD phenotype, but few spoke about the cognitive strengths and difficulties of ADHD. Children and their teachers could benefit from psychoeducation, that is, understanding ADHD in more depth (i.e., broader cognitive and behavioural profiles beyond diagnostic criteria), what ADHD and any co-occurrences might mean for the individual child, and *why* certain strategies are helpful. Improving knowledge using psychoeducation is known to improve fidelity to interventions (Dahl et al., 2020; Nussey et al., 2013), suggesting that this would facilitate children and their teachers to identify effective strategies and maintain these in the long-term.

The third theme, *the role of peers*, called attention to the importance of classmates for children with ADHD, and this was recognised by both children and their teachers. As peers play a role in the learning experience for children with ADHD, it is important to ensure that children have opportunities to learn in small group contexts with their peers. This finding is supported by Vygotsky’s (1978) Zone of Proximal Development; it is well established in the literature that children can benefit from completing learning activities with a partner, especially a more able peer (Vygotsky, 1978).

### Relevance of Co-Occurrences

Co-occurring conditions are common in ADHD (Jensen & Steinhausen, 2015), and there are many instances within the data presented here that may reflect these co-occurrences, in particular, the overlap with DCD and ASD. For ADHD and DCD, the overlap is considered to be approximately 50% (Goulardins et al., 2015), whilst ADHD and autism also frequently co-occur with rates ranging from 40 to 70% (Antshel & Russo, 2019). It was not an aim of the current study to directly examine co-occurrences, however it is important to recognise their relevance when interpreting the findings. Indeed, in the current sample, scores for seven children (70%) indicated a high likelihood of movement difficulty. One child scored above the cut-off for autism diagnosis referral on the AQ-10, indicating heightened autism symptoms. Further to this, some of the discussions with children and teachers seemed to be related to DCD or autism, for example, the way that they can react in social situations, or difficulties with the physical act of handwriting. This finding feeds into the ongoing narrative surrounding heterogeneity within ADHD and individualisation of strategies to support learning. Recognising the potential role of co-occurrences should therefore be a vital part of any psychoeducation programme for children with ADHD and their teachers.

### Limitations

Whilst a strong sample size was achieved for the current study allowing for rich data to be generated, it is important to acknowledge the issue of representativeness. The heterogeneity of ADHD is recognised throughout the current study, however the current study represents only a small cohort of children and young people with ADHD and their teachers which should be considered when interpreting the findings, particularly in relation to generalisation. Future research should investigate the issues raised using quantitative methods. Also on this point of heterogeneity, although we report some co-occurring symptoms for participants, the number of co-occurrences considered here were limited to autism and DCD. Learning disabilities and other disorders may play a role, however due to the qualitative nature of this study it was not feasible to collect data on every potential co-occurrence. Future quantitative work should aim to understand the complex interplay of diagnosed and undiagnosed co-occurrences.

Furthermore, only some of the teachers of participating children took part in the study; we were not able to recruit all 10. It may be, for example, that the six teachers who did take part were motivated to do so based on their existing knowledge or commitment to understanding ADHD, and the fact that not all child-teacher dyads are represented in the current study should be recognised. Another possibility is the impact of time pressures upon participation for teachers, particularly given the increasing number of children with complex needs within classes. Outcomes leading from the current study could support teachers in this respect.

It is also important to recognise the potential role of stimulant medication. Although it was not an aim of the current study to investigate knowledge or the role of stimulant medication in the classroom setting, it would have been beneficial to record whether the interviewed children were taking medication for their ADHD at school, particularly given the evidence to suggest that stimulant medication can improve cognitive and behavioural symptoms of ADHD (Rhodes et al., 2004). Examining strategy use in isolation (i.e. with children who are drug naïve or pausing medication) will be a vital aim of future intervention work.

### Implications/Future Research

Taking the findings of the whole study together, one clear implication is that children and their teachers could benefit from psychoeducation, that is, understanding ADHD in more depth (i.e., broader cognitive and behavioural profiles beyond diagnostic criteria), what ADHD might mean for the individual child, and why certain strategies are helpful. Improving knowledge using psychoeducation is known to improve fidelity to interventions (Dahl et al., 2020; Nussey et al., 2013), suggesting that this would facilitate children and their teachers to identify effective strategies and maintain these in the long-term.

To improve knowledge and understanding of both strengths and difficulties in ADHD, future research should aim to develop interventions grounded in psychoeducation, in order to support children and their teachers to better understand *why* and *in what contexts* certain strategies are helpful in relation to ADHD. Furthermore, future research should focus on the development of a tool-kit of strategies to account for the heterogeneity in ADHD populations; we know from the current study’s findings that it is not appropriate to offer a one-size-fits-all approach to supporting children with ADHD given that not all strategies work all of the time, nor do they always work consistently. In terms of implications for educational practice, it is clear that understanding the individual child in the context of their ADHD and any co-occurrences is important for any teacher working with them. This will facilitate teachers to identify and apply appropriate strategies to support learning which may well result in different strategies depending on the scenario, and different strategies for different children. Furthermore, by understanding that ADHD is just one aspect of the child, strategies can be used flexibly rather than assigning strategies based on a child’s diagnosis.

## Conclusion

This study has provided invaluable novel insight into understanding and supporting children with ADHD in the classroom. Importantly, these insights have come directly from children with ADHD and their teachers, demonstrating the importance of conducting qualitative research with these groups. The findings provide clear scope for future research, as well as guidelines for successful intervention design and educational practice, at the heart of which we must acknowledge and embrace the heterogeneity and associated strengths and challenges within ADHD.

## Interview Schedule—Teacher

### Demographic/experience

- How many years have you been teaching?
- Are you currently teaching pupils with ADHD and around how many?
- If yes, do you feel competent/comfortable/equipped teaching pupils with ADHD?
- If no, how competent/comfortable/equipped would you feel to teach pupils with ADHD?
- Would you say your experience of teaching pupils with ADHD is small/moderate/significant?

## Psychoeducation

1. What is your understanding of ADHD/Can you describe a typical child with ADHD?
  - Probe behaviour knowledge
  - Probe cognition knowledge
  - Probe impacts of behaviour/cognition difficulties
  - Probe knowledge that children with ADHD differ from each other
  - Probe knowledge that children with ADHD have co-occurring difficulties as the norm
2. (If they do have some knowledge) Where did you learn about ADHD?
  - e.g. specific training, professional experience, personal experience, personal interest/research

## Cognitive skills and strategies

1. Can you tell me about the pupil’s strengths?
2. Can you tell me about the pupil’s biggest challenges/what they need most support with?
3. When you are supporting the pupil with their learning, are there any specific things you do to help them? (i.e. strategies)
  - Probe internal
  - Probe external
  - Probe whether they think those not mentioned might be useful/feasible/challenges
  - Probe if different for different subjects/times of the day
4. In your experience, which of these you have mentioned are the most useful for the pupil?
  - Probe for examples of how they apply it to their learning
  - Probe whether these strategies are pupil specific or broadly relevant
  - Probe if specific to particular subjects/times of the day
5. In your experience, which of these you have mentioned are the least useful for the pupil?
6. What would you like to be able to support the pupil with that you don’t already do?
  - Probe why they can’t access this currently e.g. lack of training, resources, knowledge, time

### Is there anything you would like to understand better about ADHD?

- Probe behaviour
- Probe cognition

## Interview Schedule—Child

**Script:** *We’re going to have a chat about a few different things today, mostly about your time at school. This will include things like how you get on, how you think, things you’re good at and things you find more difficult. I’ve got some questions here to ask you but try to imagine that I’m just a friend that you’re talking to about these things. There are no right or wrong answers, I’m just interested in what you’ve got to say. Do you have any questions?*

## Psychoeducation

**Script:** *First we’re going to talk about ADHD (Attention Deficit Hyperactivity Disorder).*

1. Have you ever heard of/has anyone ever told you what ADHD is?
2. (If yes) If a friend asked you to tell them what ADHD is, what would you tell them?
3. Is there anything you would like to know more about ADHD?

## Cognition/strategy use

**Script:** *Now we’re going to talk about something a bit different. Everyone has things they are good at, and things they find more difficult. For example, I’m quite good at listening to what people have to say, but I’m not so good at remembering people’s names. I’d like you to think about when you’re in school, and things you’re good at and things you are not so good at. It doesn’t just have to be lessons, it can be anything.*

- Do you like school?
- Probe why/why not?
- Probe favourite lessons
- What sort of things do you find you do well at in school?
- Is there anything you think that you find more difficult in school?
- Probe: If I asked your teacher/parent what you find difficult, what would they say?
- Probe: Is there anything at school you need extra help with?
- Probe: Is there anything you do to help yourself with that?
  **Script:** *Some people do things to try to help themselves do things well. For example, when someone tells me a number to remember, I repeat it in my head over and over again.*
- Can you try to describe to me what you do to help you do these things?

- Solving a maths problem
- Planning your writing
- Doing spellings
- Trying to remember something
- Concentrating/ignoring distractions
- Listening to the teacher
- Remaining seated in class when doing work
- Working with other children in the class

- Probe: Do you use anything in lessons to help you with your work?
- Probe: What kind of things do you think could help you with your work?
- Probe: Is there anything you do at home, such as when you’re doing your homework, to help you finish what you are doing to do it well?
- Probe: Does someone help you with your homework at home? If yes, what do they do that helps? If no, what do you think someone could do to help?

## Practical

**Script:** *In this last part we’re going to talk about your time at school.*

1. How many teachers are in your class?
2. Is there anyone who helps you with your work?

Do you work mostly on your own or in groups?
